# Intra-Host Evolution Provides for the Continuous Emergence of SARS-CoV-2 Variants

**DOI:** 10.1128/mbio.03448-22

**Published:** 2023-02-14

**Authors:** Justin T. Landis, Razia Moorad, Linda J. Pluta, Carolina Caro-Vegas, Ryan P. McNamara, Anthony B. Eason, Aubrey Bailey, Femi Cleola S. Villamor, Angelica Juarez, Jason P. Wong, Brian Yang, Grant S. Broussard, Blossom Damania, Dirk P. Dittmer

**Affiliations:** a Department of Microbiology and Immunology, The University of North Carolina at Chapel Hill School of Medicine, Chapel Hill, North Carolina, USA; b Lineberger Comprehensive Cancer Center, Chapel Hill, North Carolina, USA; c Kuopio Center for Gene and Cell Therapy, Kuopio, Finland; NIAID, NIH

**Keywords:** COVID, DNA sequencing, SARS-CoV-2

## Abstract

Variants of concern (VOC) in SARS-CoV-2 refer to viruses whose viral genomes differ from the ancestor virus by ≥3 single-nucleotide variants (SNVs) and that show the potential for higher transmissibility and/or worse clinical progression. VOC have the potential to disrupt ongoing public health measures and vaccine efforts. Still, too little is known regarding how frequently new viral variants emerge and under what circumstances. We report a study to determine the degree of SARS-CoV-2 sequence evolution in 94 patients and to estimate the frequency at which highly diverse variants emerge. Two cases accumulated ≥9 SNVs over a 2-week period and one case accumulated 23 SNVs over 3 weeks, including three nonsynonymous mutations in the spike protein (D138H, E554D, D614G). The remainder of the infected patients did not show signs of intra-host evolution. We estimate that in as much as 2% of hospitalized COVID-19 cases, variants with multiple mutations in the spike glycoprotein emerge in as little as 1 month of persistent intra-host virus replication. This suggests the continued local emergence of variants with multiple nonsynonymous SNVs, even in patients without overt immune deficiency. Surveillance by sequencing for (i) viremic COVID-19 patients, (ii) patients suspected of reinfection, and (iii) patients with diminished immune function may offer broad public health benefits.

## INTRODUCTION

The B.1.1.7 SARS-CoV-2 strain (20I/501Y.V1, S mutations N501Y, A570D, D614G, P681H, T716I, S982A, D1118H) emerged in September of 2020. It represented the first in the ongoing evolution of variants of concern (VOC) for SARS-CoV-2. Strain B.1.1.7 (alpha) arose because of long-term viral replication in an immunocompromised person (1).

VOC refers to viruses whose viral genome sequences differ from their most common recorded ancestor, typically by ≥3 single nucleotide variations (SNVs). Notably, nonsynonymous mutations disproportionately accumulate in the spike (S) glycoprotein. VOC display increased infectivity in tissue culture, increased human-to-human transmission patterns, and may (Delta) or may not (Omicron) be associated with more severe clinical outcomes (2, 3). Typically, each VOC is less susceptible to vaccine-induced antibodies, and many are resistant to therapies using monoclonal antibodies. As VOC have the potential to render public health measures and vaccine efforts less effective, it is crucial to identify situations that foster the emergence of VOC.

Variants of interest (VOI) are defined by WHO as nearly complete virus sequences with genetic changes that are predicted to change the biological properties of the viral variant and which have an epidemiological signature indicative of increasing population prevalence. VOI can be defined based on sequence alone, even if the actual virus has not been isolated in pure culture. A designation of VOI precedes the designation of VOC.

Increased transmission at the population level can be due to various viral phenotypes, such as higher genome copy numbers in nasal secretions, increased environmental stability, or better/broader receptor utilization. Other mechanisms are also possible, including the ability to maintain longer shedding periods, which we define as persistence if shedding for a period longer than 14 days upon infection. It is presently unknown how frequently new SARS-CoV-2 variants arise in persistently infected COVID-19 patients.

Case studies have documented the emergence of highly divergent variants (4–9). This suggests that intra-host evolution reflects a general mechanism for the continued emergence of highly divergent, potentially more transmissible SARS-CoV-2 variants; however, these singular events were linked to underlying clinical circumstances, e.g., known instances of severe immunosuppression, including untreated HIV-associated induced immunodeficiency (10, 11).

Case studies, by design, are susceptible to observer bias. To corroborate singular observations, we investigated 94 patients who were repeatedly PCR-positive for SARS-CoV-2 without considering their clinical history. The mean and median time between PCR tests was 27 and 23 days, respectively, with a standard deviation of 19 days. Individual timelines are provided in Fig. S1. None of the participants were vaccinated against SARS-CoV-2. Multiple showed evidence of intra-host SARS-CoV-2 evolution, including in the spike protein. On the one hand, this result is encouraging, as 90% of SARS-CoV-2 persistent infections did not lead to the emergence of genomic variants. On the other hand, this suggests that persistent infection, even in not dramatically immunocompromised patients, leads to continued local emergence of SARS-CoV-2 variants if there is high-level community transmission.

10.1128/mbio.03448-22.1 FIG S1 Timeline of received samples that were PCR tested and sequenced multiple times. The Y axis are the arbitrary labels given to the patients. The x axis represents the date the samples were received. Samples are colored by the CT value recorded from the PCR test. Any sample in grey resulted in an undetermined CT value. Samples that resulted in sequencing coverage less than 90% and greater than 90% of the SARS-CoV-2 genome are represented in circles and triangles, respectively. Any sample represented as a square indicates the sample's sequencing library resulted in no mapped reads to SARS-CoV-2. Download FIG S1, PDF file, 0.2 MB. Copyright © 2023 Landis et al. 2023 Landis et al. https://creativecommons.org/licenses/by/4.0/ This content is distributed under the terms of the Creative Commons Attribution 4.0 International license.

The rapid spread of the spike protein D614G variant, which only had a single point mutation compared to the earliest human isolate (SARS-CoV2/hu/CHN/Wuhan-Hu-1/2019), shows that novel SARS-CoV-2 variants can rapidly take over the population (12–14). This pattern repeated with each new VOC, most recently Omicron (BA.1 and its sublineages BA.2, BA.4, BA.5, etc.) (15, 16).

Sequencing surveys identify new variants regularly (17), suggesting VOC emergence is not always tied to severe and overt immunodeficiency. If, on the one hand, VOC emergence were linked to singular, low-frequency events in time and space (18), travel restrictions and strict quarantine measures would be an appropriate approach to containment. On the other hand, if VOC continuously, repeatedly, and locally emerged in all communities worldwide, they would not. In the latter case, surveillance by whole-genome sequencing and worldwide vaccination would be the more prudent course of action.

These results suggest that as much as 2% of all persistently infected COVID-19 patients develop highly divergent variants, some within 3 weeks of infection. As Omicron, compared to Delta, has a relatively short duration and milder clinical disease (3), patients with persistent Omicron infection may constitute a consistent reservoir for developing future SARS-CoV-2 variants.

## RESULTS

**FIG 1:**
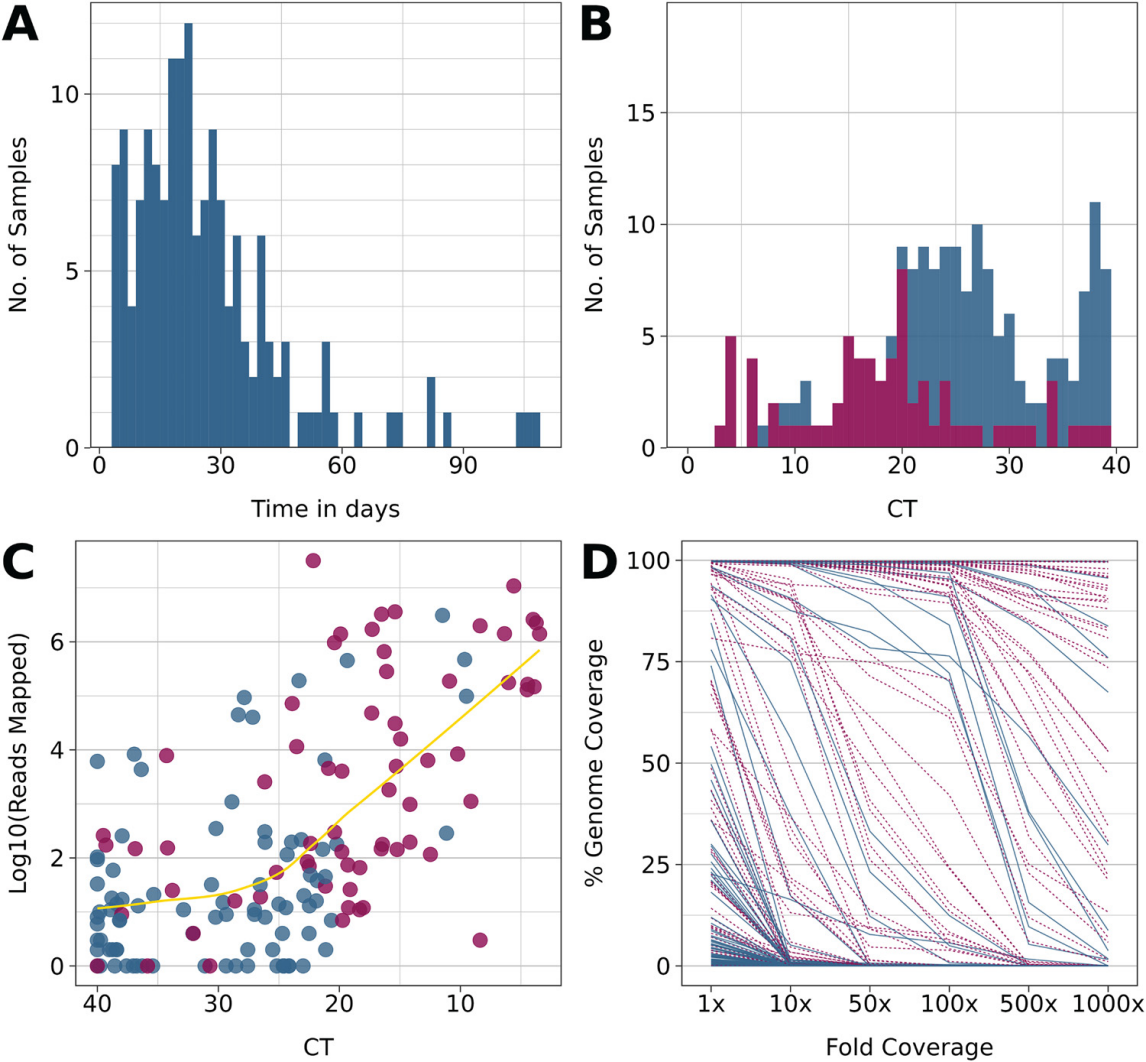
Summary characteristics of the cohort and sequencing performance. Red indicates values at baseline (T0), and blue indicates subsequent sampling points. (A) Distribution of observation time for the cohort. (B) Distribution of viral genome copy number as determined by real-time RT-qPCR. This excludes *n* = 60 samples for which no CT values were available, only a negative/positive determination. (C) Relation between genome copy number as determined by real-time RT-qPCR and fraction of reads mapped/total reads. (D) Relation between the fraction of reads mapped/total reads and coverage at 1×, 10×, and 100×.

We identified *n* = 94 cases of COVID-19 with two or more positive SARS-CoV-2 tests (Fig. 1A). The case definition included cases where intermittent viral load assays were negative, as it is not possible, *a priori*, to distinguish between persistent replication below the level of detection and independent reinfection. The median age was 52.6 ± 17.4 years (mean ± SD); 3 participants were under 18. 40/94 (43%) of the participants were female. The cases covered the period from April 1, 2020, to October 17, 2020. During that time, the COVID-19 epidemic was accelerating in the local community and was primarily driven by symptomatic transmission events (19). Vaccination coverage was limited. As most of the data were from de-identified patient records, the clinical presentations of each case could not be conclusively established. The inclusion criterion was solely based on viral detection (positive/negative) by CLIA assay in two consecutive nasopharyngeal (NP) swabs (median number of days between tests = 27 days).

All participants had detectable viral loads at baseline. At subsequent time points, the relative log_2_ viral genome copy number, as measured in the RT-qPCR cycle number threshold (CT), declined in most cases, as indicated by higher CT values (Fig. 1B). In 30 of the 94 participants, genome copy numbers at late time points were near the limit of detection (CT > 35). This was expected since even during acute symptomatic infection with SARS-CoV-2, genome copy numbers peak during a few days over the course of infection. All samples were subjected to targeted amplification of SARS-CoV-2 and next-generation sequencing (NGS) as described previously (12).

Of the 94 participants, 69 did not have high enough viral RNA levels at time points after baseline to generate enough reads to yield complete genome-wide coverage. During the time of this study, GISAID required 90% genome coverage with no guidance for a specific sequencing depth. We required coverage of 90% of the genome with a sequencing depth of > 1× overall and for SNV positions with variant quality score >200×. NGS conclusively confirmed the presence of SARS-CoV-2 RNA in all the real-time RT-qPCR positive samples and none of the RT-qPCR negative samples. The number of mapped reads showed a log-linear relationship to CT (Fig. 1C) for CT values ≤ 24.71 (^95^CI:19.11 to 33.60) and was uncorrelated for samples with CT > 24.71. As expected, more mapped reads correlated with higher overall genome coverage (Fig. 1D). These performance characteristics are in line with other studies (20).

To ascertain the linear range for SNV calling, a dilution series of SARS-CoV-2/hu/USA/WA1/2020 was generated (Table 1). Only single nucleotide variants (SNV) with variant quality scores > 200, as called by samtools (21), were included in subsequent analyses. Variant quality scores (QUAL scores) are a measure of the likelihood a called SNV arose from chance. A higher QUAL score implies that the observed SNV is unlikely to occur from chance alone (22). To estimate the sensitivity of variant calling, a dilution experiment was conducted. The SARS-CoV-2/hu/USA/WA1/2020 ATCC stock has eight SNVs compared to SARS-CoV2/hu/CHN/Wuhan-Hu-1/2019 (NC_045512). These eight SNVs were recovered over a dilution range of 4 log_10_ orders of magnitude down to a detection limit of 7 PFU/mL. The experiment was repeated with an artificial RNA substrate carrying 428-point mutations and the same dilution range. From this, 423 single-point mutations were consistently sequenced over the entire dilution range. This experiment demonstrates that the detection limit for NGS-based SNV typing is at or below the detection limit of viral culture. By implication, every complete variant sequence reported here represents a replication-competent sample rather than residual fragments of viral RNA.

**TABLE 1:** SNV calling sensitivity^*a*^

Sample	Dilution factor	IT in pfu/mL	Coverage	SNV
MT RNA (n:428)	1	NA	100.00%	426
	4	NA	100.00%	427
	16	NA	100.00%	427
	64	NA	100.00%	426
	256	NA	100.00%	427
	1,024	NA	100.00%	428
	4,096	NA	99.84%	423
	16,384	NA	98.27%	423
WT virus (n:8)	1	115,000	100.00%	8
	4	28,750	100.00%	8
	16	7,188	100.00%	8
	64	1,797	100.00%	8
	256	449	100.00%	8
	1,024	112	100.00%	8
	4,096	28	100.00%	8
	16,384	7	100.00%	8

a SNV refers to the number of correctly called SNVs relative to SARS-CoV2/hu/CHN/Wuhan-Hu-1/2019 (NC_045512) for either an artificial RNA with 428-point mutations or WT virus strain 2019-nCoV/USA-WA1/2020(BEI resources, Cat. No. NR-52281). The dilution factor shows the dilution. It refers to infectious titer in plaque-forming units/mL—coverage refers to coverage at 1×. The raw reads are available as bio project.

**FIG 2:**
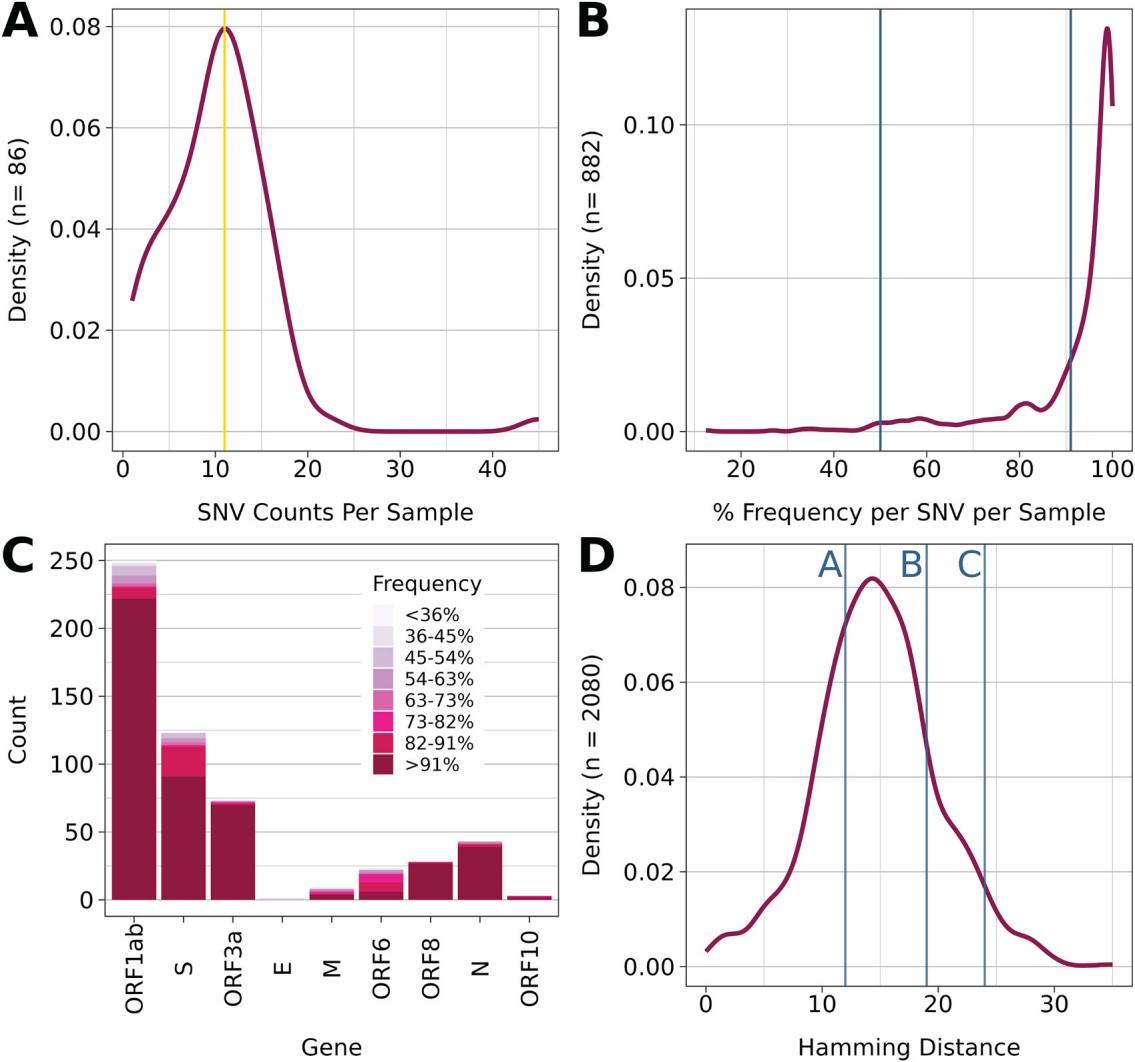
Summary of genome diversity. (A) Distribution of SNVs per sample. (B) Distribution of all SNV frequencies in the data set. (C) Count of nonsynonymous SNVs per gene, color-coded by frequency of the SNV (D) Distribution of Hamming distances of all sequenced samples that contained at least 1 SNV of Frequency greater than or equal to 51% (*n* = 65) representing 2080 comparisons. Blue lines indicated the hamming distance between paired samples. Line A represents LCCC0245 and its preceding sample of <90% coverage. Line B represents LCCC0187 and LCCC0225. Line C represents LCCC0233 and LCCC0239.

Sequence diversity in the data set is based on individual SNVs that could be ascertained with high confidence across the entire genome. This includes genes like spike that are under constant antibody selection and others that are not. A total of *n* = 882 SNVs passed the quality control filters of a QUAL score greater than 200. Figure 2A shows the average number of SNVs for each sample (*n* = 86). The mode was 11, and two samples had *n* ≥ 20 and *n* ≥ 40 SNVs. Thirty of the SNVs in the most divergent sample (>40 SNVs) existed in a frequency of >70% (The remaining samples were present at >40%). In the second-highest divergent sample, all SNVs existed in frequency >70%. While this may indicate a mixed population of viruses, these are nevertheless examples of rapid intrahost evolution of SARS-CoV-2.

Most SNVs detected had >90% frequency in the sample (Fig. 2B), although a subset of samples had additional SNVs with lower frequency. SNVs with <50% frequency were excluded from the analyses presented here, ensuring that only high confidence majority SNVs were considered. It is not possible, *a priori*, to decide whether new SNVs result from *de novo* mutation during persistence in the host or the selection of a strain that was present at a frequency below the limit of detection at the initial time point of infection. Hence, pairwise comparisons between samples represent a lower limit of sequence divergence over time.

As expected, total SNV counts correlate with the gene size. Figure 2C depicts the counts of nonsynonymous SNVs for each SARS-CoV-2 gene across all samples. Also indicated are the SNV frequencies for each gene, i.e., the proportion of SNVs with the indicated frequency per sample. As observed above, most SNVs were present at >91% frequency and would indicate the convergence of a particular SNV in all viral genotypes within the sample. This means the SNV dominates the population of the virus within the sample. Note that SNVs were present across the genome, not just in spike. This suggests mutation/selection acting on multiple genes and thus multiple steps in the viral replication cycle, including those not presently considered under immune selection.

Figure 2D shows the empirical density distribution of Hamming distances among all samples. The Hamming distance is defined as the minimum number of substitutions between any two sequences. It is less or equal to the genetic distance and is not dependent on the time or the rate of evolution. Hence, it represents a lower limit on genetic diversity. Paired samples, i.e., samples from participants with two consecutive time points that each yielded enough material for whole-genome sequencing, are indicated by blue lines. Most pairs had smaller than mean Hamming distances, consistent with the hypothesis that intra-host replication accumulates fewer mutations than inter-host transmission. Two paired samples had Hamming distances (19 and 24) that were larger than the mode, indicative of accelerated intra-host evolution during an infection period of 17 and 18 days, respectively.

On average, symptomatic COVID-19 cases are viremic for 2 weeks (23, 24). The null hypothesis stipulates that upon infection, SARS-CoV-2 replicates rapidly, synchronously, and without accumulating mutations due to the intrinsic low error rate and proofreading ability of RNA-dependent RNA polymerases of the *Coronaviridae* (25, 26) as well as limited host selection pressure prior to the onset of adaptive immunity. Consistent with this hypothesis, four pairs of participant sequences were identified with ≤ 1 SNV difference across the entire genome over 7, 10, 15, and 19 days, respectively. These represent the prototypical infection scenario for coronaviruses.

**FIG 3:**
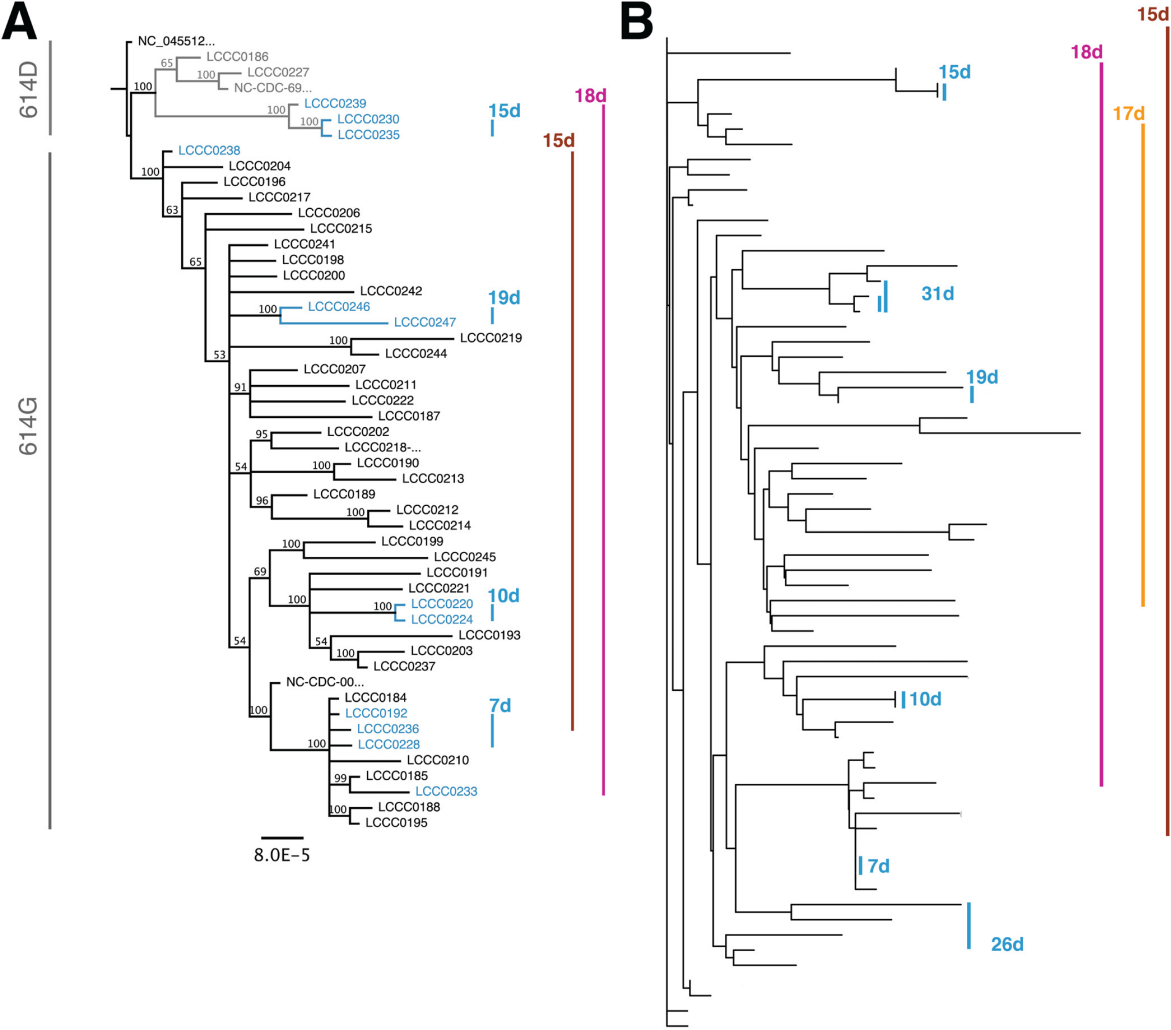
(A) Phylogenetic tree obtained from a multiple alignment *n* = 48 whole SARS-CoV-2 genomes as submitted to GISAID. The tree is under the assumption of unequal evolution rates. Paired samples are indicated by colored lines and labeled by distances in days. (B) Phylogenetic tree based on *n* = 231 high-quality SNV position for *n* = 67 complete and partial SARS-CoV-2 genomes. Paired samples are indicated by colored lines and labeled by distances in days.

Other cases accumulated more SNVs. Figure 3A depicts a phylogenetic tree obtained from the multiple alignments of *n* = 48 completely sequenced SARS-CoV-2 genomes (data available at GISAID). In addition to samples from this study, the first two SARS-CoV-2 confirmed cases in the catchment area were included: hCoV-19/USA/NC-CDC-6999/2020 from March 3, 2020 (2020-03-03), which is the spike 614D variant and hCoV-19/USA/NC-CDC-0034/2020 from March 8, 2020 (2020-03-08), which is the spike D614G variant. The reference genome SARS-CoV-2 SARS-CoV2/hu/CHN/Wuhan-Hu-1/2019 was set as root. The tree was generated from a MAFFT alignment further processed by MrBayes (HKY85 substitution model with unconstrained branch length using SARS-CoV-2 SARS-CoV2/hu/CHN/Wuhan-Hu-1/2019 as outgroup). The monophyletic sequence pairs in blue (LCCC0230/LCCC0235, LCCC0246/LCCC0247, LCCC0220/LCCC0224, LCCC0192/LCCC0228) conform to the null hypothesis of limited intra-host evolution. These pairs accumulated ≤1 SNV over a 2-week period (range: 7 to 19 days) and represent canonical infection events of a single virus.

The two sequence pairs LCCC0233/LCCC0239 and LCCC0238/LCCC236 represent cases where 24 SNVs accumulated over 18 days and 9 SNVs accumulated over 15 days, respectively. Sequencing pairs LCCC0233 and LCCC0239 were one of the two samples that displayed accelerated intra-host evolution during infection. This resulted in the sequences being distant on the phylogenetic tree. Their pattern was inconsistent with the null hypothesis and supported scenarios of accelerated intra-host evolution.

It was challenging to obtain high-quality, whole-genome sequences from samples with low genome copy numbers. These were the majority at late times after infection of an immunocompetent host (Fig. 1B and [24]). To expand the set of paired samples available for analysis, additional genomes were analyzed even if they had > 1000 N. This was possible because individual SNVs, relative to the reference, could be ascertained with high confidence (QUAL scores > 200). The use of incomplete sequences is quite common for large genomes, such as the human genome. It does not affect similarity measurements based on Hamming distance or maximum likelihood measures or GISAID clade assignments, which operate on high-quality SNV positions alone. It does interfere with timed phylogenies and recombination assessment. This yielded an extended pool of *n* = 67 samples, including the prior samples with complete genomes. Figure 3B shows a neighbor-joining tree based on this matrix and rooted at SARS-CoV2/hu/CHN/Wuhan-Hu-1/2019. The clustering by informative SNVs alone was consistent with the clustering based on analysis of the entire viral genome sequences as expected. The expanded data identified six paired samples (Fig. 3B, blue highlight), which diverged only minimally from each other during the observation period of 7, 10, 15, 19, 26, and 31 days, respectively. In contrast, 3 sample pairs were not consistent with the null hypothesis of limited intra-host evolution (Fig. 3B, purple, yellow, brown). These three pairs represent strains that have diverged significantly from the initial isolate over a period of 15, 17, and 18 days, respectively. These samples represent accelerated intra-host evolution. Note how the observation periods for the two groups overlap: over a time range of 7 to 31 days, six patients accumulated ≤1 SNV, while three patients accumulated ≥ 9 SNV over 15 to 18 days. This suggests that the conditions under which variants with multiple nonsynonymous SNVs may evolve do not necessarily require periods that are longer than 30 days, as has been previously reported in immunocompromised patients (8, 11).

**FIG 4:**
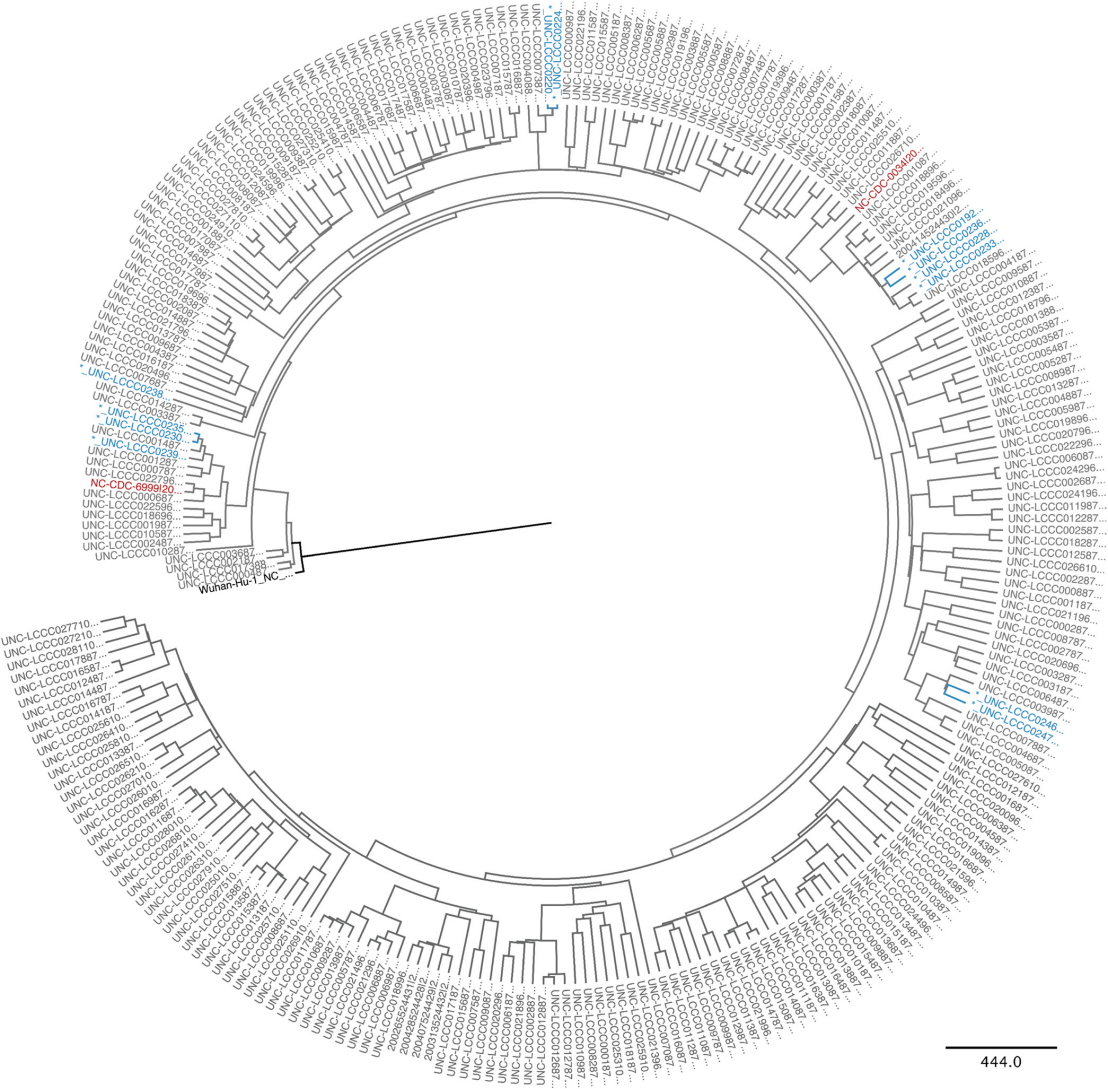
A phylogenetic tree was obtained using BEAST after a multiple alignment of *n* = 273 whole SARS-CoV-2 genomes from GISAID that were collected at the same time in the same population and determined by the same technology and bioinformatics pipeline. The initial introductory events are indicated in red, the root Hu-Wu-1 in black, and paired specimen in blue.

To test the notion that the three rapidly evolving cases perhaps started as unusual variant viruses and represented an abnormality to the local virus pool, an additional 226 whole-genome sequences from the same participant pool were generated, and a timed consensus tree was generated using BEAST (Fig. 4). The SARS-CoV2/hu/CHN/Wuhan-Hu-1/2019 genome was set as root. The two initial isolates in the state of North Carolina, one representing the D614 clade (SARS-CoV-2/human/USA/NC-CDC-6999/2020) and the other the G614 clade (SARS-CoV-2/human/USA/NC-CDC-0034/2020) were added for orientation. The resulting phylogram again identified the same four pairs as before, representing minimal intra-host evolution, as well as the two pairs that did not cluster together and represented accelerated intra-host evolution. The different paired samples belonged to different sublineages, as evidenced by their distribution among the other samples. This suggests that the higher rate of evolution observed in the three very various samples was not the property of a particular strain or a rare mutator or low fidelity polymerase variant as has been described for SARS-CoV-2 (25).

Two scenarios could account for these observations. First, in some patients, SARS-CoV-2 evolution was accelerated compared to rates determined by molecular clock phylogenies of inter-host events and the control group. This scenario also includes the sequential emergence and/or disappearance of dominant subpopulations. Second, an independent infection event, i.e., superinfection, took place prior to the last sampled time point. Sequence analysis alone cannot distinguish between these two scenarios. This study supports the notion of ongoing intra-host evolution in a significant number of SARS-CoV-2 infections that can serve as a reservoir for the continued, local emergence of highly divergent variants.

## DISCUSSION

The SARS-CoV-2 epidemic in NC was seeded by two singular events, one introducing the D614 variant strain on March 03, 2020, and another event introducing the G614 variant strain on March 13, 2020 (12). Since then, we have surveyed SARS-CoV-2 infected persons. This cohort identified 94 patients who were SARS-CoV-2 positive on at least two occasions as determined by real-time RT-qPCR under CLIA-compliant diagnostic testing. Six patients had ≤1 SNV difference for samples drawn approximately 14 days apart. Three patients had accumulated ≥1 SNV during the same time span. One participant had accumulated 23 SNVs over a 19-day period. Many of the mutations observed were concentrated in the S1 region. As opposed to the S2 domain, which is largely conserved, even in Omicron, S1 seems to be the target of neutralizing antibodies. Thus, antibody escape mutants would be expected to accumulate there (27–29).

The three participants with extensive viral evolution had potential comorbid conditions, one participant had a history of infections, and another had diabetes mellitus; however, none fit the clinical pattern of severe immunosuppression, such as that due to cancer-chemotherapy, HIV infection, or B-cell deficiency as noted in other studies (5, 8, 10, 11). Furthermore, SNVs accumulated over a much shorter time period than in these studies.

These data demonstrate that the majority of SARS-CoV-2 infections follow a paradigm of limited intra-host viral evolution consistent with the absence of selection in a naive host and are consistent with the reported mutation rate of ~1 SNV per genome per 14 days (4, 30–32).

These data demonstrate that variants with many SNVs regularly emerge in persons that carry SARS-CoV-2 as early as 2 weeks after primary exposure. By current conventions, these patients would be described as in the late acute phase or having a persistent infection. In our cohort, the average time interval between follow-up visits was 27 days, at the threshold of 4 weeks before a patient is considered to have post-Covid conditions per CDC's guidelines. 72 out of the 94 patients had symptoms greater than 14 days past initial testing, which at the time was the recommended recovery period from SARS-CoV-2 infection.

Accelerated evolution has been described for coronaviruses, e.g., those carrying a mutator polymerase or exposed to a mutating drug (25). Accelerated evolution can also be achieved by sequential bottlenecks, such as those generated during persistent low-level infection where the host immune responses dramatically reduce but never fully eradicate the virus (33–35). Even under the conservative assumptions of (a) the one most divergent sample in our study being due to reinfection with an unrelated strain and (b) the other persistently positive patients in the cohort, for which a second genome sample was incomplete, having no SNVs, we estimate that highly divergent variants of SARS-CoV-2 emerge at a frequency of 2/94 (2%) among hospitalized COVID-19 patients without symptoms of severe immunodeficiency.

There are limitations to this study. First, we could not go back and acquire additional samples or additional information due to IRB restrictions. Second, this study was conducted at the start of the COVID-19 pandemic, before any vaccination campaigns, and before the emergence of Omicron. SARS-CoV-2 Omicron infections have different clinical patterns, particularly in vaccinated individuals; however, moderately or subvert immune-challenged persons will continue to stay infected for >14 days. Some will develop suboptimal responses to vaccination. Hence, these types of longer-term acute infections will continue to occur. We submit so will the emergence of highly mutated variants at the 1/100 to 1/1,000 cases level.

This study is a single-center study biased toward patients with severe clinical diseases rather than a population-based sample. All infections were symptomatic. This contrasts with the majority of SARS-CoV-2 infected persons, who do not require hospitalization and who now have preexisting immunity.

Another limitation of this study is that many participants did not have enough viral RNA at time points subsequent to baseline for NGS to yield complete genome coverage. This was expected as, in most cases, viral RNA is detectable by a swab of the nasopharyngeal cavity (NP-swab) 2 to 3 days before the onset of symptoms (presymptomatic) but disappears within 5 days after clinical symptom onset (24, 36–39). The genome copy numbers reported here are consistent with a report of 35 RT-qPCR positive specimens collected at >10 days after symptom onset and failing to yield infectious virus upon culture (33, 40, 41). Control experiments established that complete genome coverage and confident SNV detection were possible down to a limit of 7 PFU/mL (Table 1); however, it is possible that persistent intra-host replication predominantly leads to the accumulation of fragmented viral genomes or debilitated viral particles with low transmission potential as the intra-host selection pressures differ from the selection pressures that lead to increased transmissibility between hosts. The transmission potential of these variants is unknown. We would have preferred to generate tissue culture infectious units (TCID50) for each sample. This, however, could not be done, as the samples were inactivated to allow diagnostic work.

Superinfection as a possibility can never be wholly excluded on the basis of sequence information alone, particularly in areas of high and sustained community transmission, such as during the observation period. We know that the patients were in isolation during hospitalization. Hence, a nosocomial superinfection is unlikely. We know that in 2020 SARS-CoV-2 population variation was still very limited. In 2020, the then emergent B.1.1.7 VOC only differed by 7 SNVs from the ancestorial strain. The likelihood of superinfection during the short time frame of this study, as opposed to 30 – 300 days of persistent infection that has been documented in case studies of severely immune-suppressed patients (8, 10, 11) was limited under these circumstances. If the patients followed CDC guidelines regarding quarantine and masking for patients who have been discharged, a superinfection event is less likely than intra-host evolution.

In sum, this study suggests that widely divergent SARS-CoV-2 variants, including VOI, will continually emerge spontaneously if there is significant community transmission (often stipulated as above 100/100,000 cases over a 1-week period). Intensified, repeat monitoring by sequencing hospitalized COVID-19 patients and infected at-risk persons, such as persons on immunosuppression or cancer chemotherapy (35), is helpful in identifying VOI. It may have direct clinical as well as public health benefits. This study also supports the notion that wide-scale vaccination efforts are needed globally to lower the spread of SARS-CoV-2 in the human population and thus prevent the emergence of new VOC.

## MATERIALS AND METHODS

### Resource availability.

**(i) Lead contact.** Further information and requests for resources and reagents should be directed to and will be fulfilled by Dirk Dittmer (dirkdittmer@me.com).

**(ii) Materials availability.** Full-length SARS-CoV-2 genomes that met the confidence and quality criteria detailed below were uploaded to GISAID. Other sequences, including index cases, were obtained through GISAID.

### Experimental model and subject details.

**(i) Sample collection and deidentification.** This study used remnant samples of universal transport media (UTM) from provider-collected deep nasopharyngeal (NP) swabs after their clinical purpose had been completed. The SARS-CoV-2 status of each sample was determined at The University of North Carolina at Chapel Hill Medical Center (UNCMC) McLendon Clinical laboratories. None of the samples had identifiers other than the testing date, age, and sex. Sample use was approved under human subjects' approvals number 20-2448 and number 13-2140 by the Institutional Review Board (IRB) at the University of North Carolina; CB 7097, 720 Martin Luther King, Jr. Blvd. Bldg. # 385, Second Floor, Chapel Hill, NC 27599-7097.

### Method detail.

**(i) RNA isolation.** RNA was isolated using a Magnapure24 (Roche Inc.) instrument and kits according to the manufacturer's protocol. In brief, 200 μL of UTM were neutralized with the addition of 0.1% Triton X-100 (proteomics grade, VWR: 97063-864) and 1× phosphate-buffered saline (PBS, Life Technologies, Catalog number 14190-144) to a final volume of 1.0 mL. Samples were incubated at room temperature for 30 min in a barcoded 2.0 mL screw cap tube (Roche, 07857551001), vortexing every 5 min for 15-s pulses. Using the total nucleic acid extraction protocol, the solution was processed through the Magnapure24 instrument (Roche, 07658036001). Carrier RNA (Macherey-Nagel, 740514) was added to a final concentration of 9 ng/μL. A negative reagent control and a negative cell pellet control were used for each processing batch. The reagent control consisted of 250 μL of 1× PBS instead of 250 μL of the sample in UTM. The 100 μL of purified RNA was processed for sequencing and viral load as described below.

**(ii) Real-time qPCR.** Relative viral genome copy number and cycle thresholds (Ct) were ascertained by real-time qPCR using primers and procedures previously published (42) and a protocol previously described (12). In brief, 30 μL input RNA was subjected to random hexamer-primed reverse transcription using the High-Capacity cDNA Reverse transcription kit (Applied Biosystems, 4368814). 9 μL cDNA was used for qPCR containing 125 nM for each primer and SYBR green as the detection method on a Roche LC480II Lightcycler, and Ct values were determined by an automated threshold method.

**(iii) Next-generation sequencing.** Amplicon-based next-generation sequencing was performed using a SARS-CoV-2 Ampliseq kit (ThermoFisher). We used Genomic RNA from SARS-CoV-2, Isolate USA-WA1/2020, as a positive control (BEI Resources, NIAID, NIH: NR-52285). All samples were sequenced using random hexamer/oligonucleotide dT priming according to the manufacturer's protocol on an Ion Torrent Chef (ThermoFisher 4484177) using Ion S5 Chef Solutions (ThermoFisher A27754). Samples were then loaded onto the IonTorrent S5 sequencer (ThermoFisher A27212) and 530 Chip (ThermoFisher A27763). The amplicons are tightly tiled and overlapping. Amplicon sizes ranged between 68 and 232 nucleotides after trimming low-quality sequences (Q20) and primer sequences (125-275 before trimming).

**(iv) Bioinformatic analysis.** Following primer trimming according to the manufacturer's recommendations, additional custom steps were added. Specifically, all sequences were quality trimmed using the bbduk script (arguments: qtrim=rl trimq = 20 maq = 20 minlen = 40 tpe tbo) from bbmap version 37.36. The trimmed reads were mapped to the SARS-CoV-2 reference sequence (Accession: NC_045512) using bbmap. From each mapping, the following was collected: sequence variants, mapping coverage, and a consensus sequence. Sequence variants were called from the mapping file using samtools and bcftools (21). Mapping coverage was generated using 'Deeptools' bamCoverage (43). Sequence variants and mapping coverage were used to build the consensus sequences using bcftools. Only variants with a reported QUAL greater than 200 were included in the consensus, and any region with 0× coverage was masked with Ns inserted for ambiguity. All consensus sequences derived from this study were curated to revert poly-nucleotide-tract mutations to the reference sequence. Lineages were assigned using Pangolin v.2.0 (44). Complete genomes have been submitted to GISAID, and raw reads to SRA archives. Nomenclature as per International Committee on Taxonomy of Viruses (ICTV) (45).

**(v) Phylogenetic reconstruction.** The alignments of complete genomes for this study were performed using MAFFT (46), and the initial phylogenetic tree based on whole viral genomes was generated using MrBayes (47) or RaXMAL (48) as implemented in Geneious Prime 2021.0.3. using the HKY85 substitution model and gamma-distribution-based nucleotide rate variation with unequal branch lengths. NC_045512 was used as an outgroup.

For alignment of all NC sequences as obtained from GISAID, MAFFT, and RAxML or FastTree was used for initial alignment. The alignment was exported and used as input for a time-scaled Bayesian Tree generated using BEAST v1.10.4 with the BEAGLE v3.1.0 library program (49). Estimated base frequencies using the Gamma distribution site model were selected. The coalescent exponential growth rate was selected for the previous tree using a random starting tree and a strict molecular clock. The Markov Chain Monte Carlo (MCMC) chain length was set to 10,000,000 steps, sampling after every 1000 steps (5–7). Trees were annotated using TreeAnnotator v1.10.4 and viewed on FigTree v1.4.4. Trace files were viewed on Tracer v1.7.1, and all ESS parameters were > 200. The neighbor-joining tree based on SNVs was generated using the R *phangorn* library (50) based on hamming distances calculated using the R *1071* library. All other visualizations and calculations were using R version 4.0.2 (2020-06-22).

### Quantification and statistical analysis.

Further statistical analysis and visualization were conducted using R v 4.0.0. The code is available on bitbucket.

### Data availability.

All sequence mapping algorithms and codes are publicly accessible, elaborated in detail below, or available using the CLC Genomics Workbench V 2.0 (Qiagen). R code used for data analysis is located in an accessible bit bucket folder https://bitbucket.org/dittmerlab/intermittent_covid_unc/src/master/. Alignments, analyses, and statistical groups were made as previously described by (12).
