# Supplementary material for: Intra-Host Evolution Provides for the Continuous Emergence of SARS-CoV-2 Variants
Source: mBio. 2023 Feb 14;14(2):e03448-22. doi: 10.1128/mbio.03448-22 (PMC10127995; doi:10.1128/mbio.03448-22)
Supplement: FIG S1 [file mbio.03448-22-s0001.pdf]

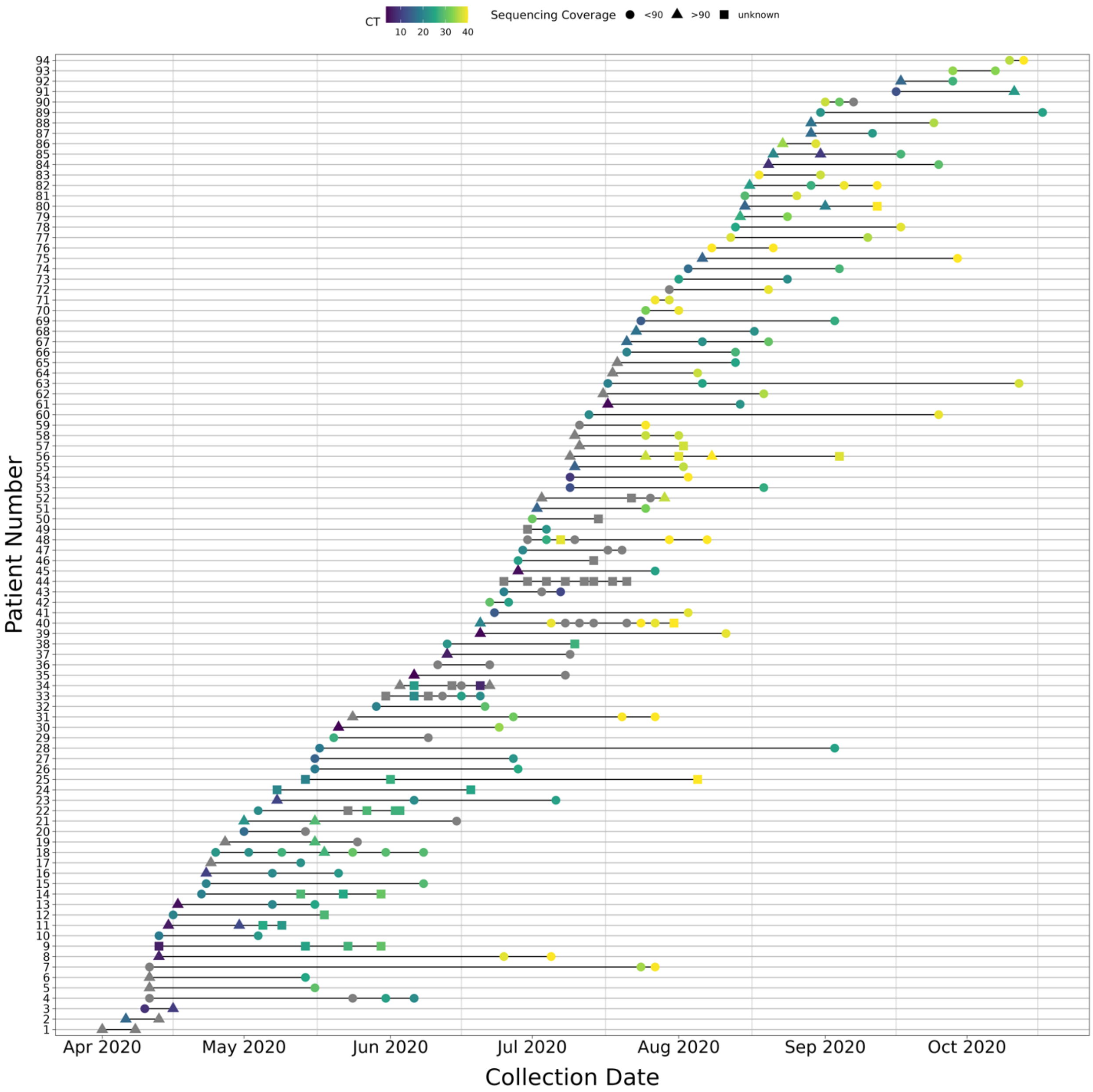

Supplemental Figure 1:

Timeline of received samples that were PCR tested and sequenced multiple times. The Y axis are the arbitrary labels given to the patients. The x axis represents the date the samples were received. Samples are colored by the CT value recorded from the PCR test. Any sample in grey resulted in an undetermined CT value. Samples that resulted in sequencing coverage less than 90 % and greater than 90% of the SARS-CoV-2 genome are represented in circles and triangles respectively. Any sample represented as a square indicates the sample's sequencing library resulted in no mapped reads to SARS-CoV-2.
